# Differentially Methylated DNA Regions in Monozygotic Twin Pairs Discordant for Rheumatoid Arthritis: An Epigenome-Wide Study

**DOI:** 10.3389/fimmu.2016.00510

**Published:** 2016-11-17

**Authors:** Anders J. Svendsen, Kristina Gervin, Robert Lyle, Lene Christiansen, Kirsten Kyvik, Peter Junker, Christian Nielsen, Gunnar Houen, Qihua Tan

**Affiliations:** ^1^The Danish Twin Registry, Epidemiology, Institute of Public Health, University of Southern Denmark, Odense, Denmark; ^2^Department of Medical Genetics, Oslo University Hospital, University of Oslo, Oslo, Norway; ^3^Denmark and Odense Patient data Explorative Network (OPEN), Institute of Clinical Research, Odense University Hospital, University of Southern Denmark, Odense, Denmark; ^4^Department of Rheumatology, Odense University Hospital, University of Southern Denmark, Odense, Denmark; ^5^Department of Clinical Immunology, Odense University Hospital, Odense, Denmark; ^6^Department of Clinical Biochemistry and Immunology, Statens Serum Institute, Copenhagen, Denmark; ^7^Unit of Human Genetics, Department of Clinical Research, University of Southern Denmark, Odense, Denmark

**Keywords:** rheumatoid arthritis, DNA methylation, monozygotic twins, epigenome-wide association study, pathway analysis

## Abstract

**Objectives:**

In an explorative epigenome-wide association study (EWAS) to search for gene independent, differentially methylated DNA positions and regions (DMRs) associated with rheumatoid arthritis (RA) by studying monozygotic (MZ) twin pairs discordant for RA.

**Methods:**

Genomic DNA was isolated from whole blood samples from 28 MZ twin pairs discordant for RA. DNA methylation was measured using the HumanMethylation450 BeadChips. Smoking, anti-cyclic citrullinated peptide antibodies, and immunosuppressive treatment were included as covariates. Pathway analysis was performed using GREAT.

**Results:**

Smoking was significantly associated with hypomethylation of a DMR overlapping the promoter region of the *RNF5* and the *AGPAT1*, which are implicated in inflammation and autoimmunity, whereas DMARD treatment induced hypermethylation of the same region. Additionally, the promotor region of both *S100A6* and *EFCAB4B* were hypomethylated, and both genes have previously been associated with RA. We replicated several candidate genes identified in a previous EWAS in treatment-naïve RA singletons. Gene-set analysis indicated the involvement of immunologic signatures and cancer-related pathways in RA.

**Conclusion:**

We identified several differentially methylated regions associated with RA, which may represent environmental effects or consequences of the disease and plausible biological pathways pertinent to the pathogenesis of RA.

## Introduction

Rheumatoid arthritis (RA) belongs to the group of complex autoimmune diseases mediated by interactions between genetic and environmental exposures. Several genome-wide association studies (GWAS) have been undertaken. Around 60 risk alleles for RA have been identified, and it is anticipated that currently known genetic risk factors only account for 16% of the total susceptibility (1, 2). However, heritability estimates from RA twin studies vary considerably from 12 to 60% (3–5). In addition, there is evidence that only comparisons between dizygotic (DZ) and monochorionic monozygotic (MZ) twins are valid for inference of genetic heritability in classical twin studies, because dichorionic MZ twins are not only identical with regard to DNA sequence but also have a higher intraclass correlation of DNA methylation (DNAm) than both monochorionic MZ twins and DZ twins (6). Therefore, higher concordance in MZ twins may not only be due to a higher degree of DNA sequence similarity since molecular mechanisms of heritability may not be limited to the DNA sequence. In addition, the diverse clinical manifestations of RA within MZ pairs concordant for RA indicate the importance of non-genetic factors on the expression of the disease (7). Thus, there is mounting evidence that environmental factors, or stochasticity, play a strong role in the etiology and expression of RA and that the effect may be mediated through epigenetic mechanisms (6, 8). Thus, to identify environmentally induced DNAm changes associated with RA, we have therefore taken advantage of the disease-discordant identical twin design to adjust for most genetic effects and many non-genetic effects such as early environment, maternal-, age-, sex-, and cohort effects. Smoking is the hitherto strongest environmental risk factor associated with RA, and in particular in the subset of RA patients possessing anti-citrullinated protein antibodies (ACPA) (9). In addition, there is strong evidence to suggest that smoking is associated with changes in DNAm (10), and genome-wide methylation data obtained on DNA from peripheral blood leukocytes suggest the existence of dynamic, site-specific methylation changes in response to smoking, which may contribute to the extended risks associated with cigarette smoking that may persist many years after cessation (11). For these reasons we have included both smoking and the presence of ACPA as covariates in the data analysis.

Inflammatory arthritis may be associated with global genomic DNA hypomethylation and with specificity for some blood cell subpopulations that is reversed with methotrexate (MTX) treatment. These changes are accompanied by parallel changes in the levels of enzymes involved in methylation, suggesting the possibility of regulation at this level (12, 13). Treatment of RA patients with MTX has also been shown to regulate defective Treg cell function through demethylation of specific genetic loci (13). As we have investigated twins with established disease, who are currently or previously treated with disease-modifying anti-rheumatic drugs (DMARDs), we have included current DMARD treatment as a covariate in the data analysis. To our knowledge, the effect of other conventional DMARDs on DNAm has not been investigated. A recent study has suggested that DNA methylation profiling may provide a new biomarker of response to biologics (14), but so far, there is no evidence to suggest that biologics themselves have a direct effect on DNAm.

Epigenetics comprise a wide range of regulatory mechanisms including histone modification, miRNA expression, and DNAm. DNAm is under constant environmental influence, highly dynamic, and differs between cell-types (15). In RA, global DNA hypomethylation has been observed in both synovial fibroblasts (RASF) (12, 16–18), peripheral blood mononuclear cells (PBMCs), and in specific subsets of T- and B-lymphocytes that may be reversed by treatment (19). Several studies have focused on DNAm of candidate loci in PBMC from RA patients, while only few studies have included multiple loci or at the epigenome-wide level (20). We therefore performed an explorative epigenome-wide association study (EWAS) characterizing DNAm differences in PMBCs from RA discordant MZ twin pairs in order to identify potential genetically independent DNAm marks associated with RA.

## Materials and Methods

### Twins

Recruitment of 28 MZ twin pairs discordant for RA was done as previously described (4, 21). The median discordance time was 18 years (interquartile range 11–30 years). RA was classified according to the ACR 1987 criteria (22). Absence of RA was verified in the co-twins based on clinical examination. Zygosity was confirmed by genetic markers (23). DNA was extracted from EDTA blood and kept at −80°C until use. RA characteristics: females 78% mean age at disease onset 38 years, anti-CCP antibody positive 61%, ever smokers 69% (smoking information missing in 14%). Sixty-eight percent were currently treated with DMARD of which 80% were treated with MTX, and none were treated with biologics or steroids. Most of the twins were in clinical remission, and the average CRP value was 3.9 mg/ml, range 0–29.

### DNAm Measurements

Genomic DNA from peripheral blood was bisulfite converted using the EZ DNA methylation Kit (ZYMO research), and DNAm status was assessed using the Infinium 450 K HumanMethylation BeadChip (Illumina) according to the manufacturer’s instructions at the Norwegian Microarray Consortium in Oslo. In order to minimize the batch effect on intra-pair DNAm differences, co-twins were processed together on the same chip. Data normalization was done using the free R package minfi, which employs subset quantile within-array normalization (24). The level of DNAm was summarized by calculating the “beta” value defined by the Illumina’s formula as β = M/(M + U + 100). We also performed QC using minfi to calculate the detection *p*-value defined as the proportion of control probes, which have intensities greater than that probe on the same array. A β value with its assigned detection *p*-value >0.01 was treated as missing. CpGs with more than 5% missing data were dropped from the subsequent analysis.

To adjust for differences in cell type composition between co-twins, we applied a statistical algorithm integrated in minfi (25, 26). All downstream analyses were based on this cell type adjusted dataset.

### Statistical Analysis

#### Identification of Differentially Methylated Positions

To identify the differentially methylated positions (DMPs) associated with RA, we fitted a linear regression model (27) to predict the mean fold change in DNAm between co-twins discordant for RA at each CpG site with adjustment for age, sex, smoking, anti-CCP antibody, and current DMARD treatment. In this model, association with RA is indicated by an intercept α that is statistically different from 0 with α > 0 for increased and α < 0 for decreased methylation levels in diseased versus healthy co-twin. A slope parameter for smoking, anti-CCP antibody, or DMARD that is statistically above or below 0 indicates exposure associated hypermethylation (β > 0) or hypomethylation (β < 0). Genome-wide significance of DMPs was determined after correcting for multiple testing by calculating the false discovery rate (FDR) with a threshold of 0.05. DMPs reaching an uncorrected *p-*value <5 × 10^−5^ were defined as suggestive.

#### Identification of Differentially Methylated Regions

Differentially methylated regions (DMRs) were identified by the free R package bumphunter (28). First, we calculated the 99th percentile of the smoothed βs to obtain upper and lower thresholds. These thresholds were then used to define hypermethylated or hypomethylated DMRs with smoothed peaks above or below the thresholds defined as putative DMRs. For each putative DMR identified, bumphunter calculates a sum statistic by taking the sum of the absolute values of all the smoothed βs within that region. The sum statistic was then used to rank all DMRs with the top-most important DMRs having the highest sum statistic value. To determine the statistical significance of each putative DMR, we performed 1000 permutations of case–control status and estimated random DMRs for each permutation. Empirical genome-wide *p*-values were calculated based on family-wise error rate (FWER) that computed, for each observed DMR-area, the proportion of maximum area values per permutation that are larger than the observed area. A DMR reaching an empirical *p*-value <0.05 was defined as significant. We defined an observed DMR as suggestive if its area was larger than the smallest area in the 1000 maximum areas from each permutation. In addition to the empirical genome-wide *p-*value, we also estimated the empirical uncorrected *p*-value for a single DMR as the proportion of all random DMRs from 1000 permutations that are larger than the area of the observed DMR. Multiple testing was corrected for by calculating FDR to obtain genome-wide significance defined as FDR < 0.05. The results from this latter method were consistent with the results based on FWER. We present the mean fold change in RA twins compared to co-twins adjusted for age, sex, smoking, anti-CCP antibody, and DMARD treatment as well as the fold change in RA twins predicted by each of the covariates smoking, anti-CCP antibody, and DMARD treatment.

### Gene-Set Analysis

We applied the DMRs as input genomic regions to Genomic Regions Enrichment of Annotations Tool [(GREAT) – version 2.0] to analyze the functional significance of *cis*-regulatory regions (29). Genome Reference Consortium Human Build 37 (GRCh37) was used as RefSeq database. GREAT was run against a whole genome background, and it performed both the binomial test over genomic regions and the hypergeometric test over genes to provide an accurate picture of annotation enrichments for genomic regions. We only present pathways with a fold enrichment of at least two by either test that are also significant at an FDR of 0.05 by both tests.

## Results

### DNAm in MZ Twins Discordant for RA

**Figure 1:**
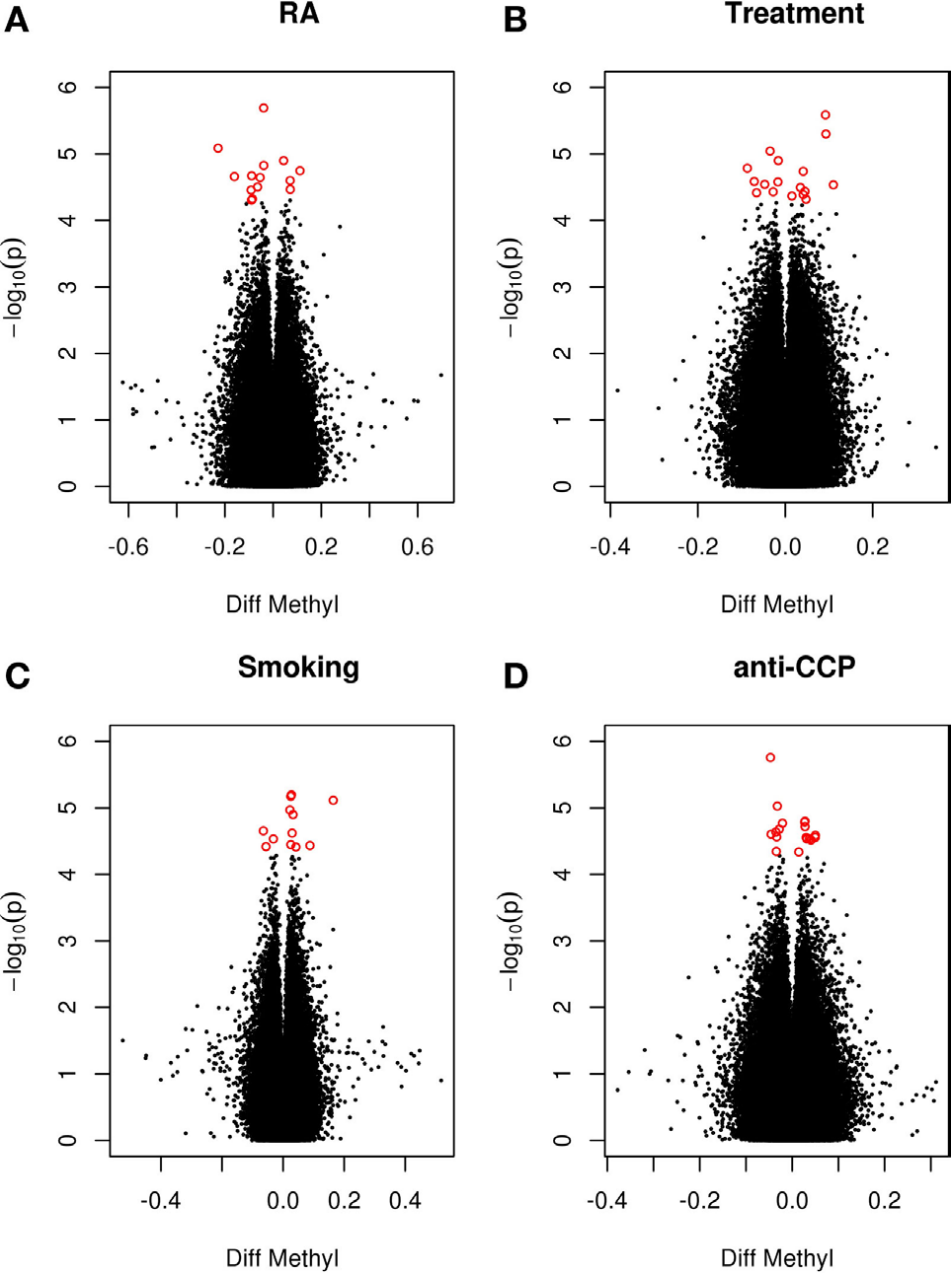
**RA-associated DMPs**. Volcano plot of detectable genome-wide differentially methylated CpG sites in 28 monozygotic RA discordant twin pairs adjusted for cell type. The −log values represent the logarithm with base 10 of the *p*-value against the methylation difference (beta values) between the RA twin and the non-RA co-twin. Positive values indicate hypermethylation in the RA twin compared to the co-twin. The red dots represent suggestive DMPs with a nominal *p*-value <5 × 10^−5^. **(A)** RA twin versus co-twin adjusting for smoking, anti-CCP antibody, and treatment. **(B)** RA twin versus co-twin predicted by smoking adjusting for anti-CCP antibody and treatment. **(C)** RA twin versus co-twin predicted by treatment adjusting for smoking and anti-CCP antibody. **(D)** RA twin versus co-twin predicted by anti-CCP antibody adjusting for smoking and treatment.

Since smoking is associated with both extensive changes in DNAm (10) and with anti-cyclic citrullinated peptide antibodies (anti-CCP antibodies) (9), smoking and anti-CCP antibodies were included as covariates. Further, MTX may elicit profound effects on DNAm (12). Since 68% of the patients were treated with DMARDs (80% with MTX), current treatment with DMARD was also included as a covariate. Analysis of these data revealed no genome-wide significant DMPs associated with RA or any of the covariates between co-twins (Figure 1).

### Regional Analyses Identify Differentially Methylated Regions

It has been reported that DNAm levels are strongly correlated across the genome and functionally relevant findings have in general been associated with genomic regions rather than single CpGs (30). The hitherto largest EWAS in RA singletons reported clustering of the most significant CpGs in regions and supports the use of region-based statistical approaches (31). Regional analysis is also less prone to be affected by the technical artifacts associated with individual probes. We therefore also performed regional analyses (i.e., “bump hunting”) (28), which allow effective modeling of measurement error and biological variability. We identified 603 putative DMRs (“bumps”) associated with RA adjusted for the other covariates. The unadjusted raw beta values of the six top ranked DMRs are presented in Figure S1 in Supplementary Material. In addition, we investigated the interaction between RA and each of the covariates smoking, anti-CCP antibodies, and treatment and identified 702, 570, and 906 putative DMRs, respectively. The top ranked DMR (364 bps) associated with RA reached borderline genome-wide significance after permutation test (*p* < 0.07). This DMR was hypomethylated with a mean fold change of 0.33 and located in the promoter region of the *S100A* (S100 calcium-binding protein A6) (Table 1; Table S1 in Supplementary Material).

**Table 1:** **The top-ranked DMRs associated with RA**.

RA and covariates	Genomic location	Distance bps	Nearest gene(s) in region	Fold change^a^	FDR^b^
**RA**					
	chr1: 153508511-153508875	364	S100A6	0.33	0.07
**Smoking^c^**					
	chr6: 32145071-32146779	1708	RNF5	0.59	0.001
			AGPAT1		
	chr4: 103940711-103941205	494	No nearby gene	0.51	0.082
	chr12: 3862221-3862497	276	EFCAB4B	1.31	0.082
**Treatment^d^**					
	chr6: 32145071-32146779	1708	RNF5	1.53	0.001
			AGPAT1		
	chr19: 9785295-9786115	820	ZNF562	0.70	0.088
	chr6: 30434072-30434552	480	No nearby gene	1.29	0.088
	chr3: 146261941-146262761	820	No nearby gene	1.78	0.092

*RA, rheumatoid arthritis*.

*^a^Fold change between RA twin and healthy co-twin. Values below or above one reflect relative hypomethylation and hypermethylation, respectively*.

*^b^False discovery rated computed using permutation testing (*n* = 1000)*.

*^c^Ever smoking versus never smoking*.

*^d^Current treatment with disease-modifying anti-rheumatic drugs*.

The region comprises a CpG island, and data from the Encode Project point to the presence of both multiple transcription factor binding sites, and a large DNAse hypersensitivity region. Furthermore, two nearby regions showed enrichment of histone marks, which indicate a regulatory role for this region. *S100A6* belongs to a cluster of genes on chromosome 1q21 encoding S100 proteins localized in the cytoplasm and/or nucleus of a wide range of cells.

**Figure 2:**
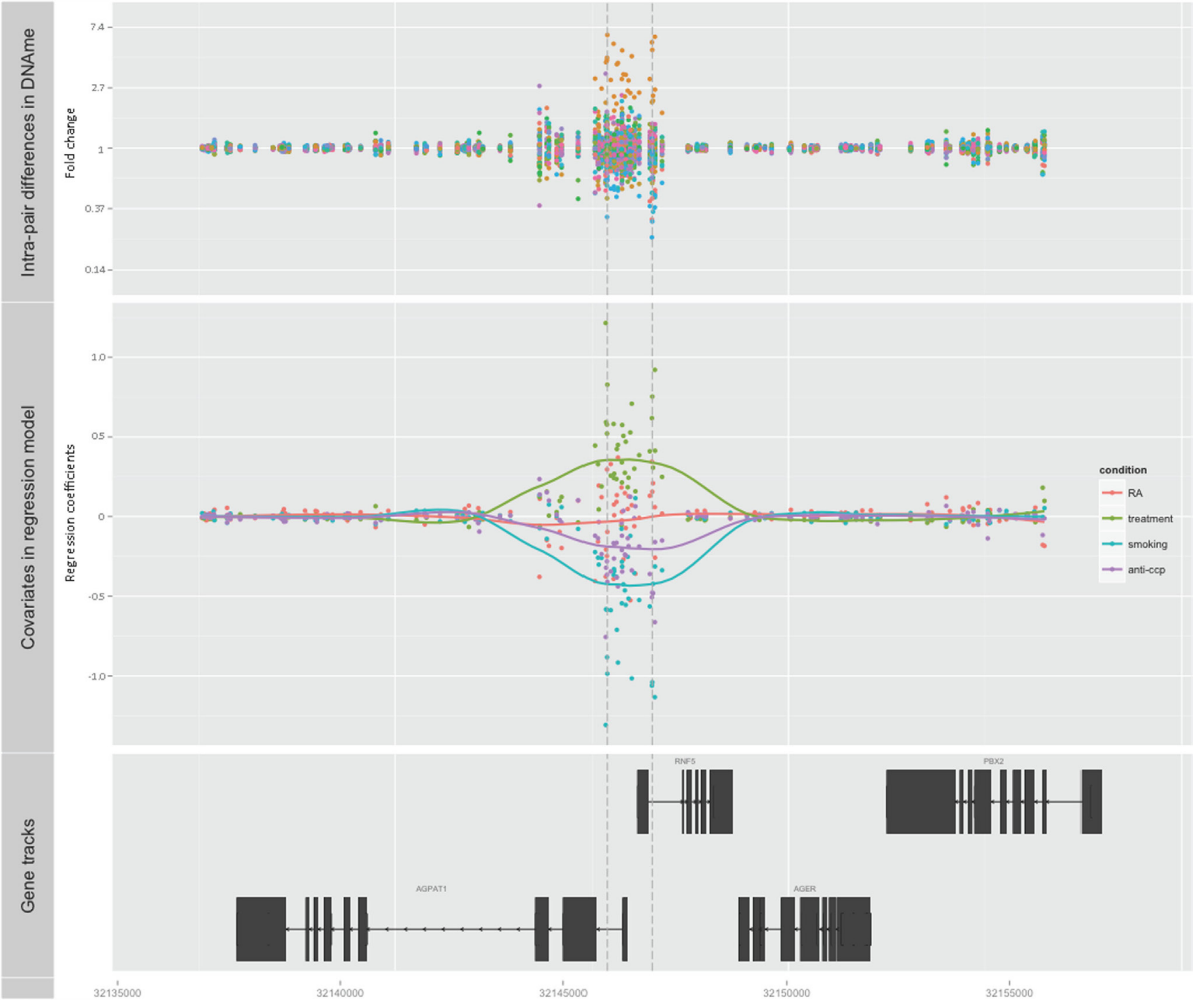
**Top-ranked DMR associated with RA**. The genome-wide significant differentially methylated region is located at the promoter region of AGPAT1 and RNF5. The top panel shows the unadjusted fold change on the *y*-axis (ratio of beta values, RA twin numerator, and co-twin denominator) of the 28 twin pairs. The middle panel displays the regression coefficients for RA (orange) and each of the covariates treatment (green), smoking (blue), and anti-CCP antibody (purple). The corresponding smoothed lines denote mean methylation level. The bottom panel depicts the genes and their genomic location.

Subsequently, we searched for DMRs associated with RA and predicted by any of the three covariates. The top ranked DMR (1708 bps) associated with RA and predicted by smoking reached genome-wide significance (*p* < 0.001). This region overlaps with the promoters of both *RNF5* (ring finger protein 5) and *AGPAT1* (1-acylglycerol-3-phosphate O-acyltransferase 1 located in the class III region of the human major histocompatibility complex) (Figure 2; Table 1; Table S1 in Supplementary Material). This region also contains several transcription factor binding sites, DNAse hypersensitivity sites, and enrichment of histone marks suggesting a regulatory role for this region. Notably, this region also reached genome-wide significance (FDR adjusted *p* < 0.001) with treatment as predictor, but with reversed DNAm pattern to hypermethylation (Figure 2; Table 1; Table S1 in Supplementary Material). This suggests that smoking, which is the strongest known environmental risk factor for RA, induces hypomethylation in this promoter region and that DMARD treatment may reverse this. It is notable that 35 of 36 CpGs were hypomethylated in RA twin modulated by smoking, and 36 of 36 CpGs were hypermethylated in RA modulated by treatment (Table S2 in Supplementary Material).

The second and third ranked DMRs predicted by smoking reached borderline significance, *p* < 0.08 (Table 1; Table S1 in Supplementary Material). There are no nearby genes within the second DMR, but the third co-localizes with a CpG island covering parts of 5′-UTR and the first exon of *EFCAB4B* (EF-hand calcium binding domain 4B).

The second ranked DMR (*p* < 0.09) predicted by treatment included the upstream region and the first part of *ZNF562* (zinc finger protein 562) and also harbors a CpG island and other elements suggestive of regulatory functions.

The top ranked DMR predicted by anti-CCP antibodies overlaps with the promotor region of two genes; *CRYZ* (Quinone oxidoreductase) and *TYW3* (tRNA-yW synthesizing protein 3 homolog), but this region did not reach genome-wide significance (*p* < 0.2).

No independent population of RA discordant twin pairs was available for replication. We therefore compared our results with the hitherto largest EWAS based on peripheral blood from anti-CCP antibody positive and treatment naïve RA singletons by Liu et al. (31). This study also corrected for cell type heterogeneity, age, gender, and smoking but did not investigate the individual effect of the covariates. These authors searched for DNAm associated with RA at the single-CpG-site level and not at the regional level, and they reduced the number of interrogated CpGs from 450 to 300 K. Consequently, a comparison at the CpG level was not optimal, and we therefore searched for replication at the gene-level among the genes associated with DMRs predicted by RA. Among our nine top ranked DMRs, we identified six nearby genes suggestively associated with RA. These genes were also reported by Liu et al. (Table 2; Table S1 in Supplementary Material), but only *S100A6, C13orf38*, and *SDCCAG1* exhibited on average the same direction of methylation.

**Table 2:** **Validation of genes nearby differentially methylated CpG sites identified in EWAS of case–control study in singletons**.

Gene^a^	EWAS singletons DMPs ref. #			EWAS MZ discordant twins		

	# CpGs^b^	Average β^c^	Bonferroni adj. *p*-value^d^	# CpGs^b^	Mean fold change^e^	*p*-value^f^
S100A6	1↓	−0.010	1.3E − 07	12↓ 3↑	0.3267	0.0678
TRIM68	1↑ 1↓	0.0014	9.98E − 09 to 1.60E − 07	12↓ 1↑	0.3516	0.9517
UNC45A	4↑	0.008375	8.21E − 10 to 7.97E − 15	18↓ 9↑	0.5915	0.9517
GPR19	2↑	0.0197	4.5E − 19 to 3.3E − 8	11↓ 6↑	0.3930	0.9517
C13orf38	1↑ 1↓	−0.00065	2.12E − 10 to 7.45E − 9	16↓ 2↑	0.6598	0.9517
SDCCAG1	4↑	0.01375	3.28E − 10 to 1.68E − 8	2↓ 12↑	1.8819	0.9517

*^a^Nearby genes defined by Illumina Annotation files*.

*^b^Number of CpGs associated with the gene. Arrow down means hypomethylation, arrow up hypermethylation for each CpG*.

*^c^Negative β means that the average value of the implicated CpG sites is hypomethylated and vice versa*.

*^d^The range of Bonferroni adjusted p-values for the CpGs*.

^e^Values below one means that the region is hypomethylated, above one hypermethylated. Fold change based on CpGs annotated to this gene and not the DMR detected by “bump hunter.”

*^f^FDR-adjusted p-values for region*.

By contrast, the two CpGs linked to *TRIM68* and *C13orf38* showed opposite directions of methylation in the study by Liu et al., whereas 12 of 13 and 16 of 18 CpGs in our study had the same direction of methylation (Table 2). Clearly, this illustrates the strength of the regional approach and indicates that these genes on average are associated with hypomethylation in RA. In total, 36 genes were overlapping the DMRs suggestively associated with RA in our data set. According to the study by Liu et al., all 36 of these genes were covered by from 1 to 7 CpGs reaching genome-wide significance in their study, but we cannot compare the direction of association beyond the 6 genes mentioned above because Liu et al. did not investigate the effect of the covariates. Interestingly, the promoters of the *RNF5* and *AGPAT1* genes were within 100-kb distance of the identified DMPs in the study by Liu et al.

### Gene-Set Analysis

We then performed GSAs to explore the potential of shared biological functions and pathways among the identified DMRs. The 603, 702, 570, and 906 putative DMRs predicted by RA, smoking, anti-CCP antibody, and treatment, respectively, comprised the input genomic regions applied to GREAT (29) to compute ontology term enrichment and identify processes or pathways that are perturbed in established RA. In Table S3 in Supplementary Material, we present the entire list of significant ontology terms and pathways. Genes with promoter regions containing the binding site for ELK1 were enriched in RA (binomial FDR 1.2 × 10^−11^) as well as in RA predicted by treatment (binomial FDR 2.7 × 10^−15^). ELK1 is a member of the E-twenty-six (ETS) oncogene family (32) and is an intracellular transcription factor of the p38MAPK signaling cascade involved in inflammation and tissue destruction in RA (33). It binds to three sites in the promoter region of tumor necrosis factor alpha (TNF-α) (34), a key player in the inflammation of RA. Genes upregulated in cervical cancer, thyroid carcinoma, and breast tumor were enriched using the RA dataset, and genes upregulated in breast and ovarian cancer were enriched in RA predicted by smoking. Gene sets that represent cell states and perturbations within the immune system were also enriched.

A key assumption of GSA requires that all genes, *a priori*, have the same probability of appearing. In cases, where some genes are tested many more times than others, genes with more associated probes are more likely to fulfill whatever *ad hoc* criteria to define differentially methylated genes. This may cause a strong bias and as the Illumina 450 K BeadChip contains from 1 to 1288 probes per gene, this type of bias should not be neglected. However, the GSA presented in this study is based on regions, which may average out the number of probes per region and thereby mitigating this bias and reduce the number of spurious findings. Thus, we did not find any correlation between the significance level of the pathways and the number of probes per gene and the most significant pathways clustered around the mean number of CpGs per gene for the whole microarray (Figure S2 in Supplementary Material).

## Discussion

This is the first comprehensive EWAS in MZ twins discordant for RA. We did not disclose DMPs associated with RA but identified one genome-wide significant DMR and several candidate DMRs. EWAS in MZ discordant twin pairs are particularly useful to detect DMRs caused by environmental or stochastic effects as compared with findings from case–control studies in singletons, which are also influenced by genetic variation. Some of our top ranking DMRs located in the promoter region of genes have previously been reported in a large EWAS on RA singletons (31). Thus, replication of these findings in a different setting of MZ twin pairs discordant for RA significantly adds to the evidence that DNAm changes in these genes may mediate the effect of environmental exposures, including drug treatment.

The strongest signal was observed in the promoter region of *RNF5* and *AGPAT1*. The protein encoded by *RFN5* is an 18-kDa RING finger membrane-bound ubiquitin E3 ligase, which has not previously been associated with RA (35). It has been shown to decrease the level of autophagy. It has been reported previously that macrophages from RNF5 knock-out mice contain a greater number of autophagosomes surrounding bacterial pathogens than wild-type mice, and this RNF5 is also implicated in antiviral innate immune signaling (36). RNF5 has been shown to be downregulated in patients with established Crohn’s disease and ulcerative colitis (37) as well as in patients with spondyloarthritis and chronic gut inflammation (35). Increased expression of this gene has been reported in PBMCs from RA patients with active disease (38), and SNP genotypes of both *RNF5* and *AGPAT1* have been associated with susceptibility to type 1 diabetes (39). Thus, both *RNF5* and *AGPAT1* have been associated with inflammation and autoimmunity.

The *S100A6* has been shown to be overexpressed in peripheral blood from RA patients and the expression level correlated to MMP3 levels (40), which accords with our finding of hypomethylation in the promoter region. The active gene may therefore be a predictor of cartilage and bone destruction. The *S100A6* is also overexpressed in salivary glandular epithelial cells in patients with Sjögren’s syndrome (41).

EFCAB4B is a Ca(2+)-binding protein that plays a key role in store-operated calcium entry (SOCE) in T-cells (42), and five different single nucleotide variations in EFCAB4B have previously been associated with RA (43). *ZNF562* may be involved in transcriptional regulation and has to our knowledge not previously been associated with RA.

Although the DMR nearby *CRYZ* and *TYWS* did not reach genome-wide significance in our study, DMPs in the promotor regions of these genes did reach genome-wide significance in the study by Liu et al. (31) DMRs overlapping these genes have previously been related to response to biologics (44), and the genes have been associated with both inflammation and Type 2 diabetes (45).

None of the genes nearby our top ranked DMRs were among the five different genes that according to Liu. et al. (31) may mediate their effect through changes in DNAm. If the DNAm differences predominantly mediate genetic effects, we would not expect to find these DNAm differences within our RA discordant MZ twin pairs. Hence, this discordance with Liu et al. indirectly supports that the effect of these polymorphisms are mediated *via* DNAm variation.

Previous studies by Nile et al. and Ishida et al. reported hypomethylation of single, but distinctive, CpG motifs in the promoter region of interleukin-6 in genomic DNA from PBMCs, which were associated with RA (46, 47). However, both the number and position of CpG motifs in the IL-6 promoter region differed between the two populations and neither of the two distinct motifs were among the interrogated sites on the 450 K Illumina assay. Among the other five interrogated CpG motifs in the promoter region of IL-6 on the 450 K assay, none were associated with RA in our study in accordance with the findings in the UK (46) and the Japanese studies (47).

In a study by Liao et al., 10 CpG motifs in the promoter region of CD40L on the X chromosome from CD4+ T cells were found to be hypomethylated in female RA patients and correlated with mRNA expression (48). We found no aberrant methylation pattern when analyzing the same promoter region separately for male and female twin pairs. This discrepancy may be explained by genetic variation between cases and controls in the Chinese study as well as differences in ethnicity, interrogated motifs, and cell type specificity. Furthermore, differences in treatment may also contribute to divergence from their results.

Genes upregulated in poorly differentiated thyroid carcinoma compared to normal thyroid tissue were enriched using the RA dataset. Autoimmune thyroid diseases (AITD) are associated with RA, and several studies have shown an association of AITD and papillary thyroid cancer (49). A higher prevalence of papillary thyroid cancers has been reported in RA patients (50) and other systemic autoimmune disorders including Sjøgrens syndrome (51, 52). Although the increased malignancy risk in RA is primarily associated with lymphomas and lung cancer (53–55), our GSA also revealed enrichment of genes upregulated in breast cancer and ovarian cancer. Even though there in no evidence of a direct association of RA with these cancers, it has previously been emphasized that the destructive process of RA may share features with neoplastic tissue. Thus, RA synoviocytes can grow under anchorage-independent conditions with defective contact inhibition, and synovial dedifferentiation and angiogenesis and mutations in the p53 tumor suppressor gene have been described in synovial tissue (56). From an epigenetic viewpoint, cancer and RA are characterized by genome-wide hypomethylation (12, 57, 58), and the demonstration of pathways shared by cancer and RA may provide new insights into disease-overlapping aspects of these two disease categories and might help to explain the invasive aspects of the diseases.

Although our study has taken advantage of the efficient MZ co-twin control method with adjustment for important confounders, some limitations should be considered. Thus, mosaicism for *de novo* mutations, retrotranspositions, indels, duplications, and chromosomal rearrangements may play a role in MZ twin discordance. In addition, copy number variants (CNVs) may manifest as DNAm changes, as suggested in one study on autism (59), although CNVs in general seem to have little impact on bead-array-based measures of DNAm (60). Also, we can not exclude the possibility of residual confounding due to cell type heterogeneity.

The inflammatory process in RA is systemic and may beside joints target a variety of extra-articular sites. While the etiology of RA remains elusive, there is robust evidence that T cells, B cells, and proinflammatory cytokine and chemokine networks are core pathogenetic mediators in RA. However, additional peripheral blood cells are implicated as well including innate effector cells like macrophages, mast cells, dendritic cells, and natural killer cells (61). Synovitis is caused by activation of these subpopulation of mononuclear cells in conjunction with granulocytes and angiogenesis (62). Although epigenetic studies at the level of specific cellular types seem attractive, it is worth considering that in a cross-sectional design like the present, there is an inherent risk that demethylation changes co-occurring in different cell subpopulations may go unrecognized by focusing on one particular cell type or that the significance of single cell type methylation changes are overestimated. Accordingly, in this exploratory study, which relies on a strong experimental design including MZ twins who are discordant for RA, we found it appropriate to study epigenetic marks in whole PBMCs. Despite the risk of a “dilution effect” by normally methylated subsets of cells, demethylation alterations were actually detected in several candidate regions reaching genome-wide significance at *p* levels between 0.001 and 0.1. These effect sizes accord well with the so far largest EWAS on singletons, which also relied on DNA from PMBC (31).

We have not done technical verification, but investigator-initiated studies on the validity of the 450 K Illumina assay have shown robust results when compared to DNAm measurement based on sequencing (63, 64). Furthermore, we have minimized batch effects because samples from cases and controls were processed simultaneously and have been placed on the same plates. In order to reduce biological variation, blood samples were drawn simultaneously from either twin in each. Last but not least, since we are dealing with MZ discordant twin pairs, the effects of genetic variants affecting probe binding or read-out are indistinguishable between cases and controls.

Although, we did not collect samples for quantification of mRNA in whole blood, our results accord with previously published gene expression studies on *the RNF5* and the *S100A6* in peripheral blood from RA patients and patients with Sjögren’s syndrome (38, 40, 41).

We also considered treatment as a source of epigenetic modification. However, our study is based on a population of RA twins with different disease profiles, which may influence our findings (65). Notably, all our RA discordant twin pairs underwent standardized clinical examination, and we therefore have optimally validated information of treatment at the time of blood sampling. Thus, this is in fact the first study to address the effect of treatment in a EWAS. It may be argued that the study is underpowered because only 68% of the cases were currently treated with a DMARD. Nevertheless, we observed a significant genome-wide effect of treatment on DMAm, and interestingly that the effect of smoking on DNAm in RA was partially antagonized by DMARD treatment. Concerning power, it has previously been estimated that 15 MZ disease-discordant twin pairs have reasonable power to detect disease-associated DMRs (66). Our disease-discordant MZ twin design provides a perfect match for genetics, maternal, and cohort effects as well as age and sex. But in addition, we have also been able to adjust for important covariates known to influence DNAm, e.g., smoking and treatment, which further increases the power of our study.

By focusing on MZ twins, the case co-twin design is especially useful in epigenetic studies as one of the main tasks in these studies is to find environmental exposures that are associated with the observed epigenetic changes linked to disease status. In our model, we have measured the intra-pair differential DNAm as a function of environmental exposure, providing adjustment for confounding factors as fixed effects and taking into account random effects such as batch effect, arrangement of samples on the array, etc. The effects of age and sex are matched out in the co-twin design; however in epigenetic studies, this argument does not hold because within-twin pair difference in epigenetic measurement can be higher in old than in young twin pairs, and for some specific sites within-twin pairs, epigenetic difference can differ according to sex. Therefore, age and sex were included as pair-specific covariates with fixed effects and their effects adjusted (27).

This is a cross-sectional study including cases with already established disease implying that the epigenetic changes may reflect both cause and effect of chronic inflammation. Yet, secondary effects may help to further elucidate the RA pathogenesis and lead to discovery of early diagnostic and prognostic markers. Furthermore, individual epigenetic signatures that remain stable over time have been described (67) and hold promise for use at the level of the individual by analogy with genotypes. Such regions can be considered as candidates for assessment of DNAm associations with disease, whereas those that are particularly labile may be relevant when assessing epigenetic marks of, e.g., treatment effects. Further epigenetic studies replicating our findings in samples collected in early life and before clinical disease onset may help to resolve these issues.

In conclusion, this exploratory EWAS on a well-characterized sample of MZ twin pairs discordant for RA has identified candidate regions and plausible biological pathways pertinent to the pathogenesis of RA, which are not explained by genetic variation. Our study also strongly suggests that there is an interaction between important covariates, DNAm, and RA. The present data are available for application in other levels of, e.g., genomics, transcriptomic, and proteomics to enhance meta-dimensional analyses to achieve a more detailed comprehension of the RA disease pathways (68).

## Ethics

The study was approved by all the regional scientific ethics committees in Denmark (Projekt ID: S-20070088) and the Danish Data Protection board (J. nr. 2007-41-0747). We obtained informed written consent from all participants in the study.

## Author Contributions

AS conceived the study. QT, KG, RL, LC, and AS performed the analysis and interpretation of the data. GH, CN, LC, RL, KG, and KK contributed reagents/materials/analysis tools. AS, QT, KG, RL, LC, and PJ prepared the manuscript. All the authors read and approved the manuscript.

## Conflict of Interest Statement

This is an investigator initiated study, and the funding source had no role in design and conduct of the study; collection, management, analysis, and interpretation of the data; and preparation, review, or approval of the manuscript. The authors have declared that no competing interests exist.
