# Supplementary figures and images for: Differentially Methylated DNA Regions in Monozygotic Twin Pairs Discordant for Rheumatoid Arthritis: An Epigenome-Wide Study
Source: Front Immunol. 2016 Nov 17;7:510. doi: 10.3389/fimmu.2016.00510 (PMC5112246; doi:10.3389/fimmu.2016.00510)

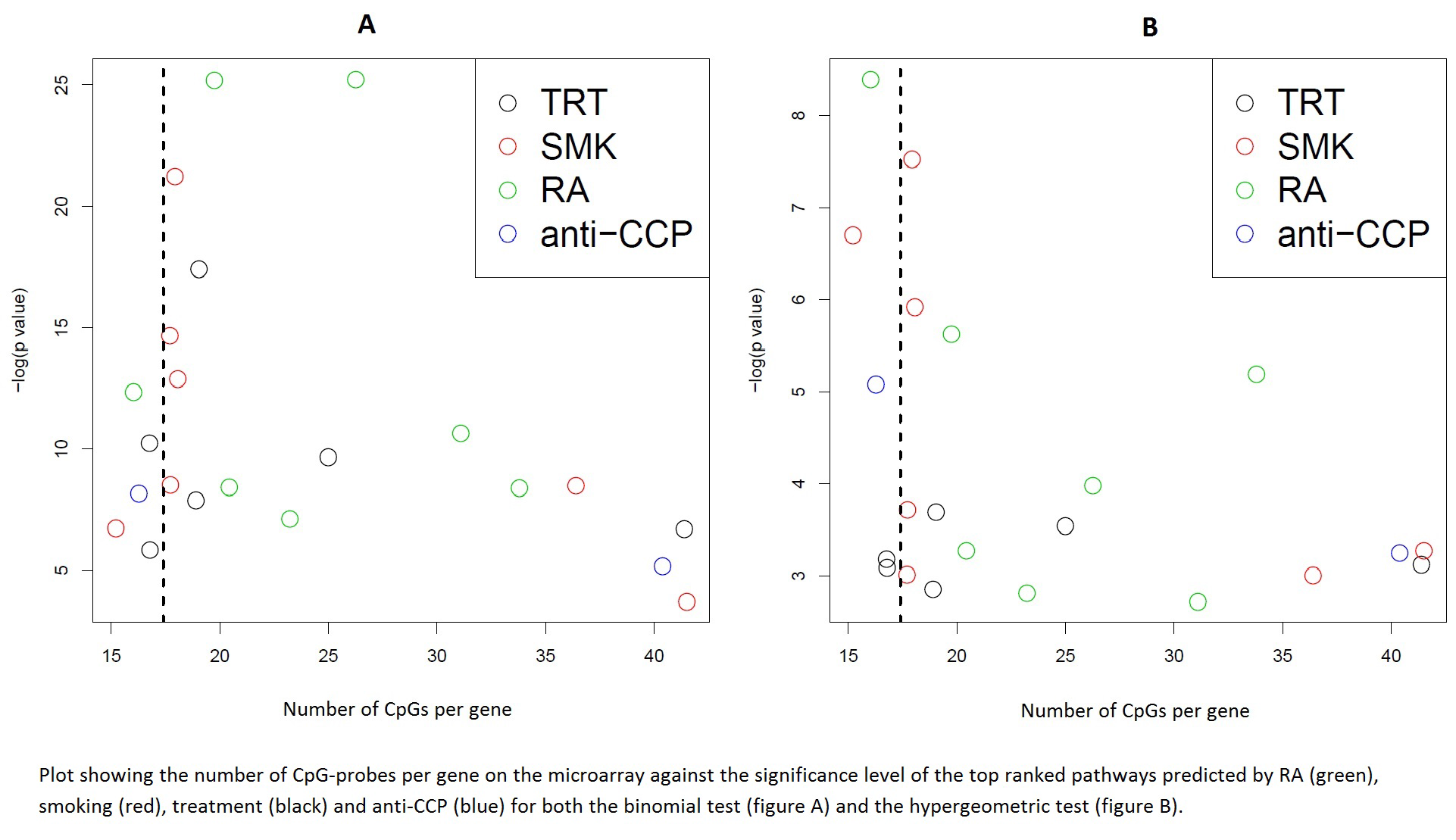

Supplement: Supplementary file 2 [file image_2.tif]
